# On the possibility of the existence of orienting hydrodynamic steering effects in the kinetics of receptor–ligand association

**DOI:** 10.1007/s00249-023-01653-0

**Published:** 2023-05-12

**Authors:** Jan M. Antosiewicz

**Affiliations:** Division of Biophysics, Institute of Experimental Physics, Faculty of Physics, University of Warsaw, Pasteura 5, 02-093 Warsaw, Poland

**Keywords:** Ligand association with a binding site, Long-range interactions, Preorientation in complex formation, Brownian motion, Rate constant

## Abstract

In the vast majority of biologically relevant cases of receptor-ligand complex formation, the binding site of the receptor is a small part of its surface, and moreover, formation of a biologically active complex often requires a specific orientation of the ligand relative to the binding site. Before the formation of the initial form of the complex, only long-range, electrostatic and hydrodynamic interactions can act between the ligand approaching the binding site and the receptor. In this context, the question arises whether as a result of these interactions, there is a pre-orientation of the ligand towards the binding site, which to some extent would accelerate the formation of the complex. The role of electrostatic interactions in the orientation of the ligand relative to the binding site of the receptor is well documented. The analogous role of hydrodynamic interactions, although assessed as very significant by Brune and Kim (PNAS 91, 2930–2934, (1994)), is still debatable. In this article, I present the current state of knowledge on this subject and consider the possibilities of demonstrating the orienting effect of hydrodynamic interactions in the processes of receptor–ligand association, in an experimental way supported by computer simulations.

## Introduction

The first step of many biological processes, at the molecular level, is the formation of a diffusional encounter complex between receptor and ligand molecules (McCammon and Harvey 1987). The created receptor–ligand complex is called a diffusional encounter complex, because its formation occurs as a result of the diffusion of receptor and ligand molecules. The encounter itself is a prelude to further processes, such as a chemical reaction, when the receptor is an enzyme and the ligand is its substrate.

The diffusive movements of molecules in a solvent are always accompanied by their long-range mutual interaction, called hydrodynamic interaction. Hydrodynamic interactions result from the fact that each moving solute particle sets the solvent medium in motion, and the resulting flux in the solvent medium then tends to move all of the other solute particles.

The induction of motion of the solvent by the surrounding molecules changes the velocity relative to the solvent of all molecules moving in it, affecting the forces and torques exerted by the solvent on the molecules. The forces and the torques may be evaluated approximately on the basis of bead models of the molecules, with each bead acting as a frictional center (de la Torre and Bloomfield 1981).

The drag force ${\textbf{F}}_i$ experienced by a bead *i* can be calculated based on the knowledge of the correction in the speed $\delta {\textbf{v}}_i$ relative to the medium, which must be added to the speed ${\textbf{v}}_i$ relative to the medium that it would have if there were no other beads

1 $$\begin{aligned} {\textbf{F}}_i = - \pi \eta \sigma \left( {\textbf{v}}_i -\delta {\textbf{v}}_i \right) . \end{aligned}$$

A correction for the relative velocity is obtained from the hydrodynamic interactions tensor ${\hat{T}}$

2 $$\begin{aligned} \delta {\textbf{v}}_i({\textbf{r}}_i) = \sum _{j \ne i} {{\hat{T}}}({\textbf{r}}_i - {\textbf{r}}_j) \cdot {\textbf{F}}_j({\textbf{r}}_j), \end{aligned}$$

where ${\textbf{r}}_i$ and ${\textbf{F}}_i$ are the position vector and friction force experienced by *i*th bead. Equation (3) shows the formula for the hydrodynamic interaction tensor ${{\hat{T}}}$, developed in 1927 by the Swedish physicist Carl Oseen (Oseen 1927)

3 $$\begin{aligned} {{\hat{T}}}({\textbf{r}}) = \frac{1}{8\pi \eta r} \left( {{\hat{I}}} + \frac{{\textbf{r}}{\textbf{r}}}{r^2} \right) . \end{aligned}$$

Rotne and Prager (1969), Yamakawa (1970), and de la Torre and Bloomfield (1977) modified the Oseen tensor to take into account the finite size of the beads.

A measure of the initial encounter complex formation rate due to reactant diffusion is the diffusional encounter rate constant, $k_{\text {d}}$. This rate constant can be determined both experimentally and theoretically. The first expression for the rate constant of diffusional encounter complex formation was introduced in 1917 by von Smoluchowski (1917). He assumed that molecules A and B are spheres with radii $\sigma _{\text {A}}$ and $\sigma _{\text {B}}$ and diffusion coefficients $D_{\text {A}}$ and $D_{\text {B}}$, respectively, and that there are no long-range interactions between them. Moreover, he assumed the existence of a stationary state in which the concentration of B molecules at “infinity” is $c_{{\text {Bo}}}$, and at the distance $R^*=\sigma _{\text {A}}+\sigma _{\text {B}}$ from the center of the molecule A is zero. This leads to the formation of a centrosymmetric concentration gradient of B molecules around the central A molecule which is the source of a constant flux of B molecules going towards the central A molecule. This flux gives the number of B molecules forming encounter complexes with A molecules per unit volume of solution and unit time

4 $$\begin{aligned} k_{\text {d}} = 4 \pi \left( D_{\text {A}}+D_{\text {B}}\right) \left( \sigma _{\text {A}}+\sigma _{\text {B}}\right) . \end{aligned}$$

In 1940, Kramers (1940) supplemented Smoluchowski’s analysis with a term related to the existence of a centrosymmetric interaction potential *U*(*r*) between associating molecules

5 $$\begin{aligned} k_{\text {d}} = 4 \pi \left( D_{\text {A}}+D_{\text {B}}\right) \left[ \int _{r=R^*}^{r=\infty } \textrm{e}^{{U}/{\textrm{kT}}}\,\frac{\textrm{d}r}{r^2} \right] ^{-1}, \end{aligned}$$

where *k* is the Boltzmann constant, and *T* is the absolute temperature.

Of the two commonly known centrosymmetric interaction potentials, the potential of electrostatic interactions is of importance for the formation of molecular complexes. Electrostatic interactions result from the fact that each molecule can be treated as a distribution of electric charges (Debye 1929). They are described by the well-known Coulomb’s law

$$\begin{aligned} U(r) = \frac{Z_{\text {A}} Z_{\text {B}} e^2}{\epsilon r}, \end{aligned}$$

where *e* is the elementary charge, $\epsilon$ is the dielectric constant of the solvent, and $Z_{\text {A}}$ and $Z_{\text {B}}$ are the electric charges of molecules A and B in units of the elementary charge. In 1942, Debye (1942) presented a form of Equation (5) for spherical particles interacting with the electrostatic potential

6 $$\begin{aligned} k_{\text {d}} = 4 \pi D_{\text {w}} \,R^*\,\frac{\frac{Z_{\text {A}} Z_{\text {B}} e^2}{\epsilon \textrm{kT}\,R^*}}{\textrm{e}^{\frac{Z_{\text {A}} Z_{\text {B}} e^2}{\epsilon \,\textrm{kT}\,R^*}-1}}. \end{aligned}$$

Then, Friedman in 1966 (Friedman 1966), using Smoluchowski’s method in the calculation, supplemented the expression derived by Kramers with the hydrodynamic interaction between spherical molecules, assuming the applicability of the Oseen tensor

7 $$\begin{aligned} k = 4\pi \textrm{kT}\left( \frac{1}{\gamma _{\text {A}}}+\frac{1}{\gamma _{\text {B}}}\right) \frac{1}{\int _{R^*}^\infty \left[ 1 + \frac{1}{4\pi \eta r \left( \frac{1}{\gamma _{\text {A}}}+\frac{1}{\gamma _{\text {B}}}\right) }\right] \frac{1}{r^2} \textrm{e}^{U(r)/\textrm{kT}}\textrm{d}r}, \end{aligned}$$

where $$\gamma _{{\text {A}}({\text {B}})}= 6\pi \eta \sigma _{{\text {A}}({\text {B}})}=\textrm{kT}/D_{{\text {A}}({\text {B}})}$$.

When molecules A and B interact with the Coulomb potential, then the integral in Eq. (7) can be solved analytically. The final result is Friedman (1966)

8 $$\begin{aligned} k = 4 \pi \textrm{kT}\left( \frac{1}{\gamma _{\text {A}}}+\frac{1}{\gamma _{\text {B}}}\right) R^* \frac{z}{\textrm{e}^z -1 + \frac{1}{4\pi \eta R^* \left( \frac{1}{\gamma _{\text {A}}}+\frac{1}{\gamma _{\text {B}}}\right) }\left[ \textrm{e}^z\left( 1-\frac{1}{z}\right) + \frac{1}{z}\right] }, \end{aligned}$$

where

$$\begin{aligned} z = \frac{Z_{\text {A}} Z_{\text {B}} e^2}{\epsilon R^* \textrm{kT}}. \end{aligned}$$

Friedman concluded his work by saying that the effect of hydrodynamic interactions on the association rate constant of spherical particles is of the order of 15% slowdown (Friedman 1966). At the same time, he noticed that the hydrodynamic effect will be present equally in the reverse reaction. Because the hydrodynamic effect cannot contribute to the free energy of an association process, it must slow down the dissociation rate by the same amount as the association.

The hydrodynamic interactions can be symbolically introduced into the rate constant formula (5) by assuming that the relative diffusion coefficient of the molecules depends on the distance between them, $D_{\text {A}} + D_{\text {B}} \equiv D_{\text {w}}(r)$

$$\begin{aligned} k_{\text {d}} = 4 \pi \left[ \int _{r=R^*}^{r=\infty } D_{\text {w}}(r) \textrm{e}^{{U}/{\textrm{kT}}}\,\frac{\textrm{d}r}{r^2} \right] ^{-1}. \end{aligned}$$

As I have presented in the abstract, the main topic of this paper is the hydrodynamic orienting effect described by Brune and Kim (1994). To the best of my knowledge, after the publication by Brune and Kim of the hypothesis on the role of hydrodynamic interactions in forcing the orientation of the ligand relative to the receptor-binding site, no one else except me and my collaborating researchers from two laboratories took up this topic. My aims in this paper is to summarize the results presented in our work and to consider the chances that such hydrodynamic effects can be demonstrated, especially in an experimental way. This review is organized as follows. First, I briefly introduce the concepts of electrostatic steering and orientational electrostatic steering, which refer to well-documented effects. Next, I introduce the concept of hydrodynamic orientational steering, primarily recalling the work of Brune and Kim and early references to their hypothesis. In the fourth part, I present an experimental attempt to demonstrate of the existence of orienting effects of hydrodynamic interactions using the well-studied process of forming complexes between lysozyme and *N*-acetyl-chitotriose. I introduce the concept of the binding anisotropy coefficient and refer to the hypothesis presented in an earlier publication that the dependence of this parameter on the viscosity of the solvent may indicate the existence of such orienting effects. The new result is to show that the equation for the association rate constant of two hydrodynamically interacting spherical molecules, derived by Friedman, leads to the viscosity-independent binding anisotropy coefficient. In the last part, I present the conclusions and possible experimental activities that could potentially confirm the presented hypothesis about the relationship between the viscosity dependence of binding anisotropy with hydrodynamic orientational steering.

## Electrostatic orientational steering

Unlike hydrodynamic interactions, electrostatic interactions can accelerate or slow down the formation of molecular complexes. The influence of electrostatic interactions on the kinetics of complex formation was called electrostatic steering. This term was used in the title of a publication for the first time in 1990 (Sines et al. 1990), but it was introduced to the scientific literature even earlier, at least in 1986 (Northrup et al. 1986). In the most common sense, the term referred to the electrostatic attraction between the ligand and the receptor, which is superimposed on the chaotic Brownian motion of the molecules. The presence of electrostatic steering in the above sense is found when increasing the ionic strength of the solvent is accompanied by a significant decrease in the association rate constant (Wallis et al. 1995; Meltzer et al. 2006; Xue et al. 2014; Huang et al. 2015; Blöchliger et al. 2015; Mohan et al. 2016; Xu et al. 2016; Huang et al. 2017; Jiang et al. 2019; Gao et al. 2021). In addition, the term “electrostatic steering” can also be understood as the influence of the moments of electrostatic forces on the orientation of the ligand relative to the binding site of the receptor. The existence of this effect was first demonstrated by Luty et al. (1993), who studied the diffusional encounter between the glycolytic enzyme triose phosphate isomerase and its substrate, D-glyceraldehyde phosphate by Brownian dynamics simulations. By reversing the direction of the dipole moment on the substrate model, they showed that the orientational steering of the substrate by electrostatic torques can significantly increase the reaction rate constant. Hemsath et al. (2005), investigating the recognition of a protein involved in the regulation of cell division called Cdc2 by Wiskott–Aldrich Syndrome Proteins using biochemical, kinetic, structural, and computational methods, concluded that an important role of electrostatic interactions is the steering of two proteins when they approach each other. The interactions accelerate their proper orientation and increase the rate of their association. Lumb and Sansom (2012), studying the membrane docking of a protein containing a membrane directing domain, called the pleckstrin homology (PH) domain, coined the term translational and rotational electrostatic steering. Orientational electrostatic steering was also described for association of hen egg-white lysozyme with $\alpha$-lactalbumin (Perrsson and Lund 2009), and for the Tet repressor protein binding to operator DNA (Porschke 2013). One of the published works even mentions about the pre-orientation of the ligand relative to the receptor-binding site by electrostatic moments of force when they are several angstroms apart (Guo et al. 2013). Thus, the existence of the orienting effect of electrostatic force moments between the associating receptor and its ligand seems well documented.

## Hydrodynamic orientational steering

At the beginning of the 1990s, the belief that electrostatic forces and moments of electrostatic forces are the second, next to diffusion, factor influencing the rate of formation of molecular complexes became established. The influence of hydrodynamic interactions was usually neglected. For this reason, it was quite a surprise to present the hypothesis of the dominant influence of hydrodynamic interactions on forcing the correct longitudinal orientation of the ligand molecule approaching the longitudinal binding cavity of the receptor.

Brune and Kim (1994) considered hydrodynamic interactions in the process of forming a complex of an elongated substrate with an enzyme having a binding site in the shape of an elongated cavity. The aim of Brune and Kim was to determine the size of the torque causing a change in the orientation of the ligand relative to the receptor-binding site resulting from intermolecular hydrodynamic interactions. They used the cleft enzymes as a model system, as they could be expected to show strong hydrodynamic effects. One particular type of hydrodynamic interaction stands out: a steering torque which occurs when the enzyme and substrate move toward each other in solution. Brune and Kim compared the magnitude of this steering torque to the mutual torque experienced by interacting “protein-sized” dipoles in solution, and concluded that the hydrodynamic steering torque can be two orders of magnitude greater than the electrostatic torque (Brune and Kim 1994).

The existence of orienting hydrodynamic effects when associating molecules approach each other requires hydrodynamic coupling of their translational and rotational movements. This coupling occurs, for example, in irregularly shaped molecules and affects their Brownian motions (Brenner 1965, 1967). Moreover, if a molecule with this coupling moves with a linear speed under the influence of an external force, the translational motion, due to the existence of the coupling, will force the molecule to rotate. In the case of a molecule association process, this external force may be electrostatic attraction by the association process partner. Strong electrostatic attraction occurs, for example, in the case of barnase and barstar associations (Schreiber and Fersht 1996). It has been shown that the approach of barstar to barnase is accompanied by a rotation leading to the crystallographic arrangement of the proteins (Długosz and Antosiewicz 2013).

The existence of hydrodynamic translation–rotation coupling in molecules of DNA with 179 bp was observed in the transients of linear electric dichroism of their solutions subjected to rectangular pulses of the electric field, analyzed on the basis of bead models of bent DNA molecules (Antosiewicz and Porschke 1989; Porschke and Antosiewicz 2005; Porschke 2007; Antosiewicz and Porschke 2009). It was the translation–rotation coupling in the DNA molecules pulled by the electric field in the measurement cell in the “field-on” phase of electro-optical experiments that resulted in such a distribution of the orientation of the DNA molecules that led to the unusual transients of the linear electric dichroism of their solutions.

Wu and Nitsche (1995), noting the detailed hydrodynamic calculations pertaining to rotational drag coefficients for a spherocylindrical “substrate” interacting with a spherical doublet “enzyme” by Brune and Kim, emphasized that these have yet to be built into a reaction–diffusion analysis. This was done by Antosiewicz and McCammon (1995). Their Brownian dynamics simulations showed that the hydrodynamic torques have only a modest effect on the preferred orientation of the ligand approaching the “enzyme”. The model used by Antosiewicz and McCammon was a little different from that used by Brune and Kim, but for the same conditions as those adopted by Brune and Kim, they obtained very similar values of the torques resulting from hydrodynamic interactions.

**Fig. 1:**
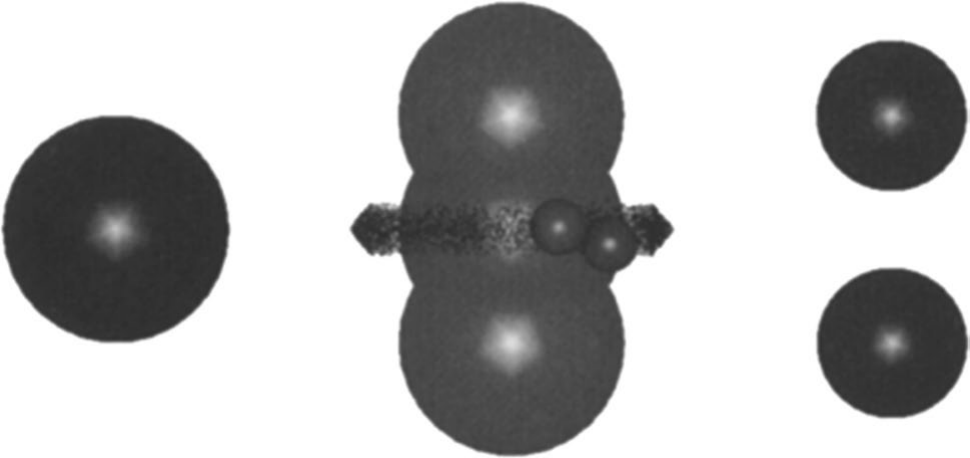
Presentation of the models used in the Antosiewicz et al. (1996). Three overlapping spheres in the central part depict the area inaccessible to the dumbbell substrate. The ring around the central sphere, marking the area inaccessible to the substrate, defines the positions in which the centers of the spherical elements modeling the substrate must be at the same time, so that the reaction criteria for the formation of the enzyme–substrate complex are met. In the central part of the figure, an exemplary location of the dumbbell substrate that meets the reaction criteria is also shown. The globular enzyme model is shown on the left side of the figure, and the enzyme model with a binding cavity in the form of two spheres with a radius of 5 A each, separated by 11 A, is shown in the right. The radius of the globular enzyme is selected, so that the average translational diffusion coefficients of both models are the same. Taken from Eur. Biophys. J., 24:137–141 (1996) with permission

In a subsequent work, Antosiewicz et al. (1996) considered the dependence of the diffusional enzyme–substrate encounter rate constants on the ionic strength of the solvent, for two types of enzyme substrate models, one in which orienting hydrodynamic effects are present and one in which these effects are absent (see Fig. 1). Both enzyme models are hydrodynamically equivalent in the sense that their average translational diffusion coefficients are the same. Both enzyme models have an electrical charge of + 10e placed at their centers. Each spherical element of the substrate model has a charge of − 2e. The beads in the dumbbell enzyme model are uncharged. Thus, the formation of the encounter complex is aided by the attractive electrostatic force directed towards the center of enzyme coordinate system.

**Fig. 2:**
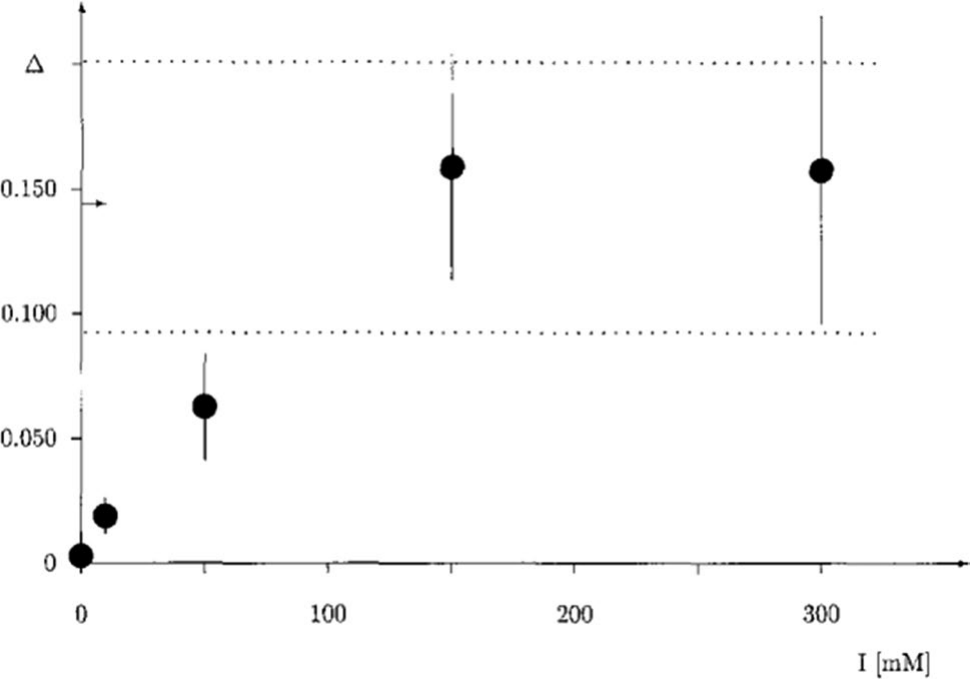
Relative increase in the diffusional encounter rate constant, $\varDelta = (k_{d,d}-k_{s,d})/k_{s,d}$, as a function of ionic strength I. The horizontal arrow on the perpendicular axis indicates the average value of $\varDelta$ for non-charged particles with the same hydrodynamic properties, and the horizontal dotted lines indicate the estimated error range for the non-charged case. Taken from Eur. Biophys. J., 24:137–141 (1996) with permission

Diffusional encounter rate constants were obtained from Brownian dynamics simulations for dumbbell substrate and dumbbell enzyme ($k_{d,d}$), and for single bead enzyme with dumbbell substrate ($k_{s,d}$), as functions of the ionic strength of the solvent. This led to parameter $\varDelta$ as a measure the complex formation anisotropy

9 $$\begin{aligned} \varDelta \equiv \frac{k_{{\text {d,d}}}-k_{{\text {s,d}}}}{k_{{\text {s,d}}}} . \end{aligned}$$

As can be seen in Fig. 2, there is a 15% change in parameter $\varDelta$ when the ionic strength grows from 0 to 150 mM, and then, it reaches a constant value. This is the result of the orienting effect of hydrodynamic interactions, leading to an increase in the constant rate of association with the assumed criteria for the formation of the encounter complex. This result gave rise to the idea that experimental studies conducted in solvents with changing ionic strength may reveal the orientation effects of hydrodynamic interactions in the processes of receptor–ligand association.

## In search of experimental evidence of the orienting effects of hydrodynamic interactions

Let us summarize our considerations so far. Brune and Kim (1994) showed that we can expect the orienting effects of hydrodynamic interactions to reveal themselves in the kinetics of the association process of molecules with appropriate hydrodynamic properties, provided that they approach each other with a sufficiently high speed. However, Brune and Kim paper greatly overestimated the steering torque, because they used the thermal velocity rather than a diffusive drift velocity. The required speed of approach of the ligand to the binding site of the receptor can only be obtained as a result of attractive electrostatic interactions. In turn, Antosiewicz et al. (1996) showed that one of the possibilities to demonstrate the existence of orienting hydrodynamic effects in the processes of molecular associations experimentally is to determine the dependence of the rate constants of the formation of diffusional encounter complexes on the ionic strength of the medium.

However, there is a problem with the experimental implementation of the idea presented by Antosiewicz, Briggs, and McCammon. In practice, it is probably impossible to prepare the enzyme–substrate system in the test and reference forms, which are satisfactorily similar in terms of the distribution of the molecular electric charge, and differ in a desirable way in terms of hydrodynamic properties. The difference is supposed to be that in the test system, there is a coupling of the translational and rotational movement of the ligand approaching the receptor-binding site, and in the reference system, there is no such coupling. Creating such a pair of models for the purposes of computer simulations does not cause any special problems.

On the other hand, actions aimed at changing the distribution of electric charge in partners in the process of association, while maintaining their hydrodynamic properties is quite possible to implement in practice. If the receptor in the association process is a protein molecule, the standard procedure is site-directed mutagenesis to create mutated proteins (Flavell et al. 1974) and the use of circular dichroism spectroscopy to verify that the mutation has not caused changes in the protein structure (Kelly et al. 2005).

**Fig. 3:**
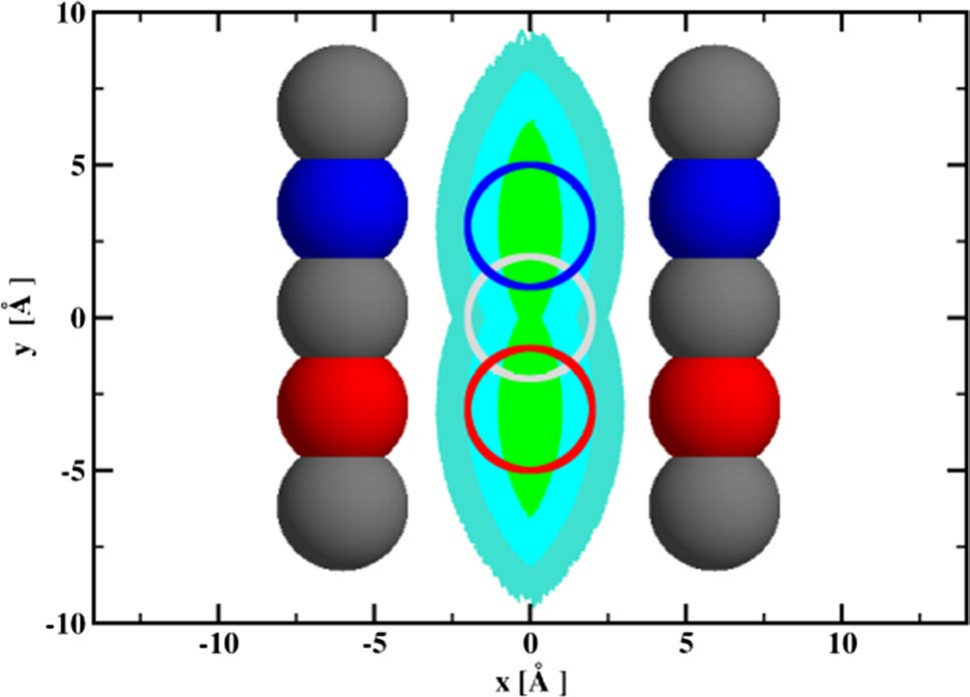
Bead model of the “wild type” elongated binding cleft and elongated ligand at the center of the binding site. The “mutated form” of the binding cleft has both red beads on the one array of the beads and the two blue beads on the other—thus, its dipole moment is perpendicular to its orientation in the “wild type”. Three gradually more restrictive diffusional encounter reaction criteria are represented as projections on the xy-plane of three-dimensional turquoise, cyan and green, respectively, volumes where the two extreme beads of the ligand model must be simultaneously located to satisfy the given reaction criterion. Less significant details in the description of the model used are provided in the original reference (Wielgus-Kutrowska et al. 2021)

This idea was implemented by Wielgus-Kutrowska, Marcisz, and Antosiewicz for the tri-*N*-acetylglucosamine-hen egg-white lysozyme system (Wielgus-Kutrowska et al. 2021). They used stopped-flow fluorimetry to determine rate constants for the formation of diffusional encounter complexes of tri-*N*-acetylglucosamine (NAG3) with hen egg-white lysozyme ($k_{\text {a}}^{{\text {WT}}}$) and its double mutant Asp48Asn/Lys116Gln ($k_{\text {a}}^{{\text {MT}}}$). Subsequently, they defined binding anisotropy

10 $$\begin{aligned} \kappa \equiv \frac{k_{\text {a}}^{{\text {WT}}}-k_{\text {a}}^{{\text {MT}}}}{k_{\text {a}}^{{\text {WT}}}+k_{\text {a}}^{{\text {MT}}}}, \end{aligned}$$

and determined its ionic strength dependence. Unfortunately, the obtained relationship was nothing like the one presented in Fig. 2, that is, there was no increase in the $\kappa$ value with the initial increase in the ionic strength followed by a plateau. Moreover, the values of the binding anisotropy $\kappa$ were rather small and noisy. Either the mutation in lysozyme did not introduce a sufficient change in the electrostatic attraction/orienting of tri-*N*-acetylglucosamine toward the binding cleft or the hydrodynamic interaction does not discriminate between ligand molecules approaching the cleft with different orientations. Due to the lack of technical possibilities to carry out extensive subsequent mutations of lysozyme, Wielgus-Kutrowska, Marcisz, and Antosiewicz decided to look for clues for the necessary modifications in the design of their experiments based on the results of simulations using Brownian dynamics methods. Figure 3 presents the model used in the simulations. Besides two variants of the electric charge distribution, named as the “wild type” and “mutated form”, two variants of Brownian dynamics simulations were performed. One with hydrodynamic interactions between all beads present in the models included and the other with neglecting hydrodynamic interactions between beads forming the binding site and beads forming the elongated ligand.

**Fig. 4:**
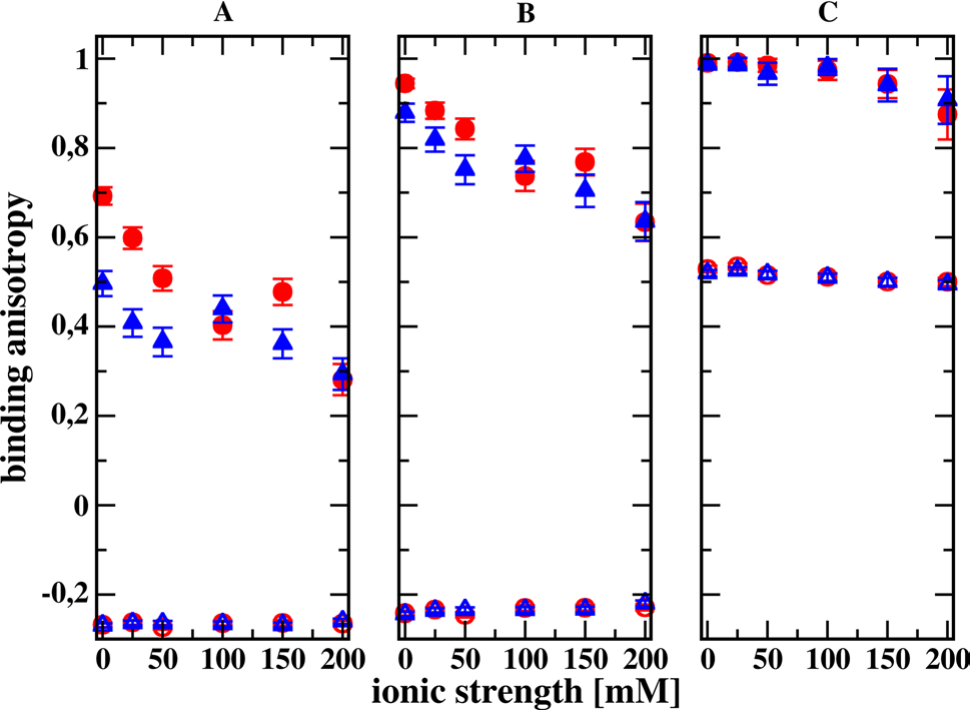
Comparison of the ionic strength dependencies of the binding anisotropy $\kappa$ defined by Eq. 10 for the three reaction criteria (A being the less restrictive and C being the most restrictive; see Fig. 3), obtained from Brownian dynamics simulations for two different solvent viscosities (1.002 cP, red; 1.150 cP, blue), with (filled symbols) and without (empty symbols) intermolecular hydrodynamic interactions included

The most important result obtained from the simulation of the association process using Brownian dynamics is shown in Fig. 4, because it suggests that the presence of hydrodynamic orienting effects can be inferred from the ionic strength dependence of $\kappa$ determined for solvents of different viscosity. As we can see, for the ionic strength below 100 mM and for both less-restrictive criteria for creating the encounter complex, we observe that the ionic strength dependencies of $\kappa$ for solvents of different viscosities are different. At higher ionic strengths, the dependencies on ionic strength for both viscosities tested are, within the statistical error, the same. For the most restrictive reaction criterion, the dependences of $\kappa$ on ionic strength are the same within the statistical error. However, it should be emphasized that the number of registered complex formation reactions for this criterion is relatively small, so the $\kappa$ values are burdened with a significant statistical error. It is also possible that the hydrodynamic interaction tensors Rotne and Prager, Yamakawa, and Garcia de la Torre and Bloomfield, used in the simulations as being just the next level approximation of the Oseen disturbance only, fail in the correct description of hydrodynamic interactions for the most restrictive reaction criterion. It should also be noted that the models of receptor and ligand molecules have a net electric charge of zero, so the electrostatic attraction is caused only by dipole interactions. The dipole moment of the wild-type receptor is 115 Debye units, and that for the model of the receptor mutant is 230 D. The first is oriented along the longer symmetry axis of the receptor, the latter is perpendicular. These values are comparable to hen egg-white lysozyme dipole moment 140–240 D, measured by dielectric relaxation methods (Bonincontro et al. 2004; Wolf et al. 1824). The ligand dipole moment is 57.6 D; therefore, it is 5–10 times larger than expected for small organic molecules. If intermolecular hydrodynamic interactions are not taken into account in the simulations, the dependences of the $\kappa$ parameter on the ionic strength for both viscosities overlap. We can see that binding anisotropies significantly decrease with increasing ionic strength of the solvent in the case of simulations taking into account receptor–ligand hydrodynamic interactions. On the other hand, when these hydrodynamic interactions are neglected in the simulations, the binding anisotropies seem to be independent of the ionic strength. This is interesting, because the individual association rate constants for both the wild-type and mutant receptor strongly decrease with increasing ionic strength. Due to the statistical dispersion present in the simulation results, it is not clear whether the independence of the binding anisotropy from the ionic strength in the case of neglecting the receptor–ligand hydrodynamic interactions is exactly fulfilled. This question could be answered by deriving an equation of the Smoluchowski type, taking into account the shielding effect of electrostatic receptor–ligand interactions by dissolved salt ions.

At this point, we can ask the question whether the differences in ionic strength dues for different viscosities, shown in Fig. 4, may indicate the existence of orienting effects of hydrodynamic interactions. The answer is probably yes, since we can expect $\kappa$ to be independent of viscosity for hydrodynamically interacting molecules when they have spherical shapes. This follows from Eq. (8) derived by Friedman (Friedman 1966). According to this equation, the association rate constant of spherical particles is the product of two terms

$$\begin{aligned} 4 \pi \textrm{kT}\left( \frac{1}{\gamma _{\text {A}}}+\frac{1}{\gamma _{\text {B}}}\right) R^* \end{aligned}$$

and

$$\begin{aligned} \frac{z}{\textrm{e}^z -1 + \frac{1}{4\pi \eta R^* \left( \frac{1}{\gamma _{\text {A}}}+\frac{1}{\gamma _{\text {B}}}\right) }\left[ \textrm{e}^z\left( 1-\frac{1}{z}\right) + \frac{1}{z}\right] }. \end{aligned}$$

The second term does not depend on viscosity, because

$$\begin{aligned} \frac{1}{4\pi \eta R^\star \left[ \frac{1}{\xi _{\text {A}}}+\frac{1}{\xi _{\text {B}}}\right] } = \frac{1}{ 4\pi \eta R^\star \left[ \frac{1}{6\pi \eta \sigma _{\text {A}}}+\frac{1}{6\pi \eta \sigma _{\text {B}}}\right] } = \frac{1}{ 2 R^\star \left[ \frac{ 1}{3\sigma _{\text {A}}}+\frac{1}{3\sigma _{\text {B}}}\right] }; \end{aligned}$$

therefore, this term only depends on the distribution of electric charges within the spherical particle. We can also transform the first term

$$\begin{aligned} 4 \pi \textrm{kT}\left( \frac{1}{\gamma _{\text {A}}}+\frac{1}{\gamma _{\text {B}}}\right) R^* = \frac{2\textrm{kT}R^*}{3\eta }\left( \frac{1}{\sigma _{\text {A}}}+\frac{1}{\sigma _{\text {B}}}\right) , \end{aligned}$$

and the equation for the rate constant of formation of encounter complexes can be presented as

$$\begin{aligned} k = \frac{2\textrm{kT}{R^*}^2}{3\eta }\left( \frac{1}{\sigma _{\text {A}}}+\frac{1}{\sigma _{\text {B}}}\right) \frac{z}{\textrm{e}^z -1 + \frac{1}{ 2 R^\star \left[ \frac{ 1}{3\sigma _{\text {A}}}+\frac{1}{3\sigma _{\text {B}}}\right] } \left[ \textrm{e}^z\left( 1-\frac{1}{z}\right) + \frac{1}{z}\right] }. \end{aligned}$$

Therefore, $k_{\text {a}}^{{\text {WT}}}$ will be different from $k_{\text {a}}^{{\text {MT}}}$ for a given viscosity $\eta$, but both will be proportional to $1/\eta$. Consequently, $\kappa$ for hydrodynamically interacting spherical molecules will not depend on the viscosity of the solvent. Such a dependence may appear only for non-spherical particles.

At this point, it is worth noting that the dependence of $\kappa$ on the viscosity comes from the fact that the association rate constants include drift velocity contributions that are proportional to $1/\eta$ and Brownian contributions that are proportional to $1/\sqrt{\eta }$. Both contributions should be significant, so that the anisotropy of the association rate constant, $\kappa$, is dependent on the viscosity of the solvent. The weakening of the electrostatic attraction with the increase of the ionic strength should lead to the disappearance of the dependence of kappa on viscosity with the increase of the concentration of salt ions shielding these interactions. This is visible in the results of simulations conducted in the ionic strength range of 0–500 mM (Wielgus-Kutrowska et al. 2021).

Certainly, further simulations are necessary to obtain possible confirmation of the hypothesis that intermolecular hydrodynamic interactions in the case of some receptor–ligand pairs, for which approaching each other causes rotation of one molecule relative to the other, can be inferred from the differences in the change of solvent viscosity, described above. Simulations, however, are computationally expensive and require careful consideration of the models for which they would be carried out. This is a matter for the future. We will end this article with the only experimental example that allows us to present the results in the form shown in Fig. 4, although the work in which we found them did not introduce the concept of anisotropy of the association rate constant as defined by Eq. (10).

**Fig. 5:**
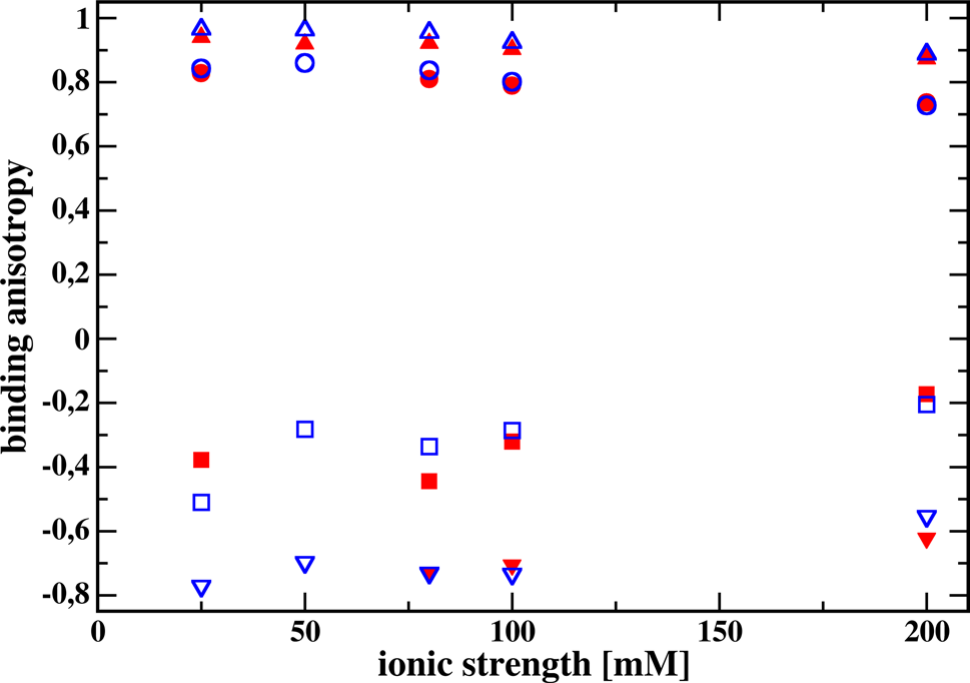
Comparison of the ionic strength dependencies of the binding anisotropy $\kappa$ defined by Eq. (10) for formation of diffusional encounter complexes of baranse wild-type wand its four mutants with wild-type barstar, with (empty symbols) and without (filled symbols) addition of glycerol to the buffer. Considered mutants of barnase: Arg59Ala (circles), Lys27Ala (triangle up), Glu73Trp (squares), and Asp54Ala/Glu60Ala (triangle down)

The only paper that determined the rate constants of wild-type and mutant protein encounter complexes for more than one solvent viscosity that we were able to find is the paper by Schreiber and Fersht describing the associations of barnase and barstar (Schreiber and Fersht 1996). Schreiber and Fersht determined the complex formation rate constants between wild-type barstar and wild-type barnase and its various mutants, as a function of ionic strength determined by the concentration of added NaCl, in buffer with and without 20% glycerol. By reading from their Fig. 2 the values of the rate constants and the corresponding ionic strengths for solutions without and with the addition of 20% glycerol, we can create a graph corresponding to our Fig. 4. All rate constants are for barstar wild type. In addition to wild-type barnase, we used the rate constants obtained for the following mutants: Glu73Trp, Asp54Ala/Glu60Ala, Arg59Ala, and Lys27Ala. Data extracted from Fig. 2 of Schreiber and Fersht (1996) are presented in the form of binding anisotropy in our Fig. 5. In this Figure we do not see a clear difference between the anisotropy values of the barnase–barstar association for both solvent viscosities. However, it should be emphasized that the values of the rate constants were read from the drawings in the original publication (Schreiber and Fersht 1996), which certainly reduces the accuracy of the calculated $\kappa$ anisotropies.

## Conclusions and possible future

In this paper, I presented a literature review describing the influence of long-range electrostatic and hydrodynamic interactions on the kinetics of the formation of diffusion complexes of receptor molecules and its ligand. The main subject of my interest was the issue of the possibility of the impact of hydrodynamic interactions on forcing the correct orientation of the ligand in relation to the shape of the binding cavity, before their surfaces become clogged. Brune and Kim (1994) called this the action of hydrodynamic steering torque.

**Fig. 6:**
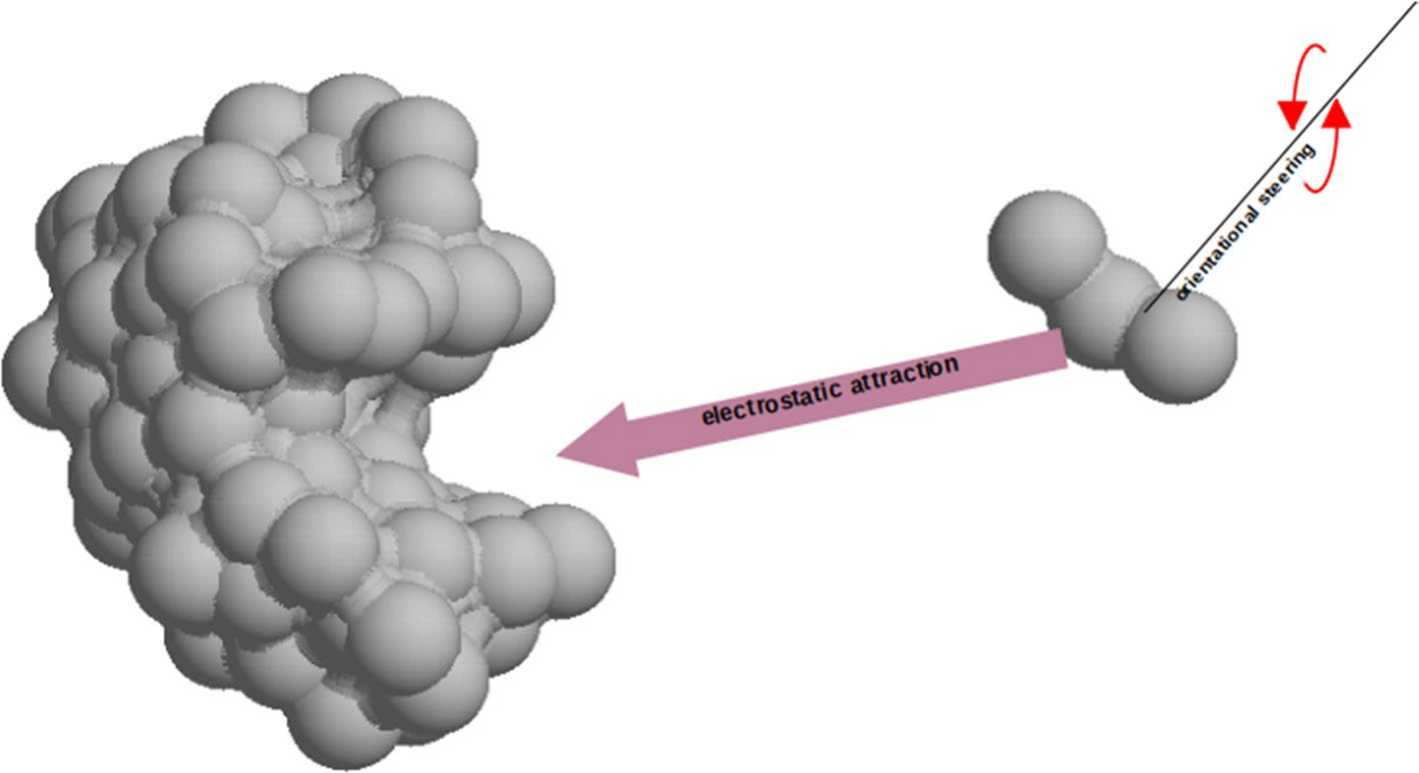
A cartoon representation of the process of forming a diffusive encounter complex between a ligand and a receptor-binding site in which the electrostatic attraction of the ligand by the receptor is accompanied by rotation of the ligand resulting from the hydrodynamic coupling of translational and rotational movements

In one of the previous papers with my co-authorship (Wielgus-Kutrowska et al. 2021), we suggested that the existence of the orienting effect of hydrodynamic interactions can be investigated by studying the kinetics of associations in solvents of different viscosities. In the present work, I showed that two hydrodynamically interacting spherical molecules would not exhibit the viscous effects described in that paper. Thus, the viscosity dependence of the anisotropy of the diffusional encounter complex formation rate constant indicates the existence of the hydrodynamic torque affecting the orientation of the ligand approaching the receptor-binding site.

Experimental demonstration of the existence of the orienting hydrodynamic torque requires a receptor molecule and a ligand molecule that meet two conditions: (1) approaching each other, they should exhibit substantial mutual coupling between their translational and rotational motions; (2) their electrostatic attraction should be sufficiently strong. This is illustrated in Fig. 6. Certainly, finding the right receptor and ligand for these studies is quite a challenge. DNA-binding proteins seem to be interesting systems to investigate in this regard. As we know, short DNA oligonucleotides are quite stiff sticks, and such studies could use native proteins binding to these nucleotides and their mutants with a changed orientation of the electric dipole moment. Due to the net negative charge of the DNA oligonucleotide and the usually positive charge of DNA-binding proteins, there should be an electrostatic attraction of these molecules, which will give the molecules a sufficiently high speed of approaching each other. Finally, we can add that even demonstrating the existence of this effect in dilute solutions does not determine its existence and significance in living systems.

## Data Availability

Original data, obtained in the author's laboratory and used in the preparation of this article, is available upon request.
